# Disruption of Methionine Metabolism in *Drosophila melanogaster* Impacts Histone Methylation and Results in Loss of Viability

**DOI:** 10.1534/g3.115.024273

**Published:** 2015-11-06

**Authors:** Mengying Liu, Valerie L. Barnes, Lori A. Pile

**Affiliations:** Department of Biological Sciences, Wayne State University, Detroit, Michigan, 48202

**Keywords:** methionine metabolism, histone methyltransferase, histone demethylase, *Drosophila*

## Abstract

Histone methylation levels, which are determined by the action of both histone demethylases and methyltransferases, impact multiple biological processes by affecting gene expression activity. Methionine metabolism generates the major methyl donor *S*-adenosylmethionine (SAM) for histone methylation. The functions of methionine metabolic enzymes in regulating biological processes as well as the interaction between the methionine pathway and histone methylation, however, are still not fully understood. Here, we report that reduced levels of some enzymes involved in methionine metabolism and histone demethylases lead to lethality as well as wing development and cell proliferation defects in *Drosophila melanogaster*. Additionally, disruption of methionine metabolism can directly affect histone methylation levels. Reduction of little imaginal discs (LID) histone demethylase, but not lysine-specific demethylase 2 (KDM2) demethylase, is able to counter the effects on histone methylation due to reduction of SAM synthetase (SAM-S). Taken together, these results reveal an essential role of key enzymes that control methionine metabolism and histone methylation. Additionally, these findings are an indication of a strong connection between metabolism and epigenetics.

Methionine is the initiating amino acid in the synthesis of virtually all eukaryotic proteins while methionine metabolism provides many metabolites important for a number of other pathways and biological processes (Brosnan and Brosnan 2006). Methionine metabolism generates the primary methyl donor *S*-adenosylmethionine (SAM) from methionine through SAM synthetase (SAM-S) (Figure 1). SAM is converted to *S*-adenosylhomocysteine (SAH) via methyltransferases by donating a methyl group to a receptor, such as DNA, RNA, histones, other proteins and smaller metabolites. SAH is hydrolyzed to homocysteine and adenosine by adenosylhomocysteinase (AHCY). Homocysteine is converted to cystathionine via cystathionine-β-synthase (CBS), or it is remethylated to methionine through methionine synthase (MS).

**Figure 1:**
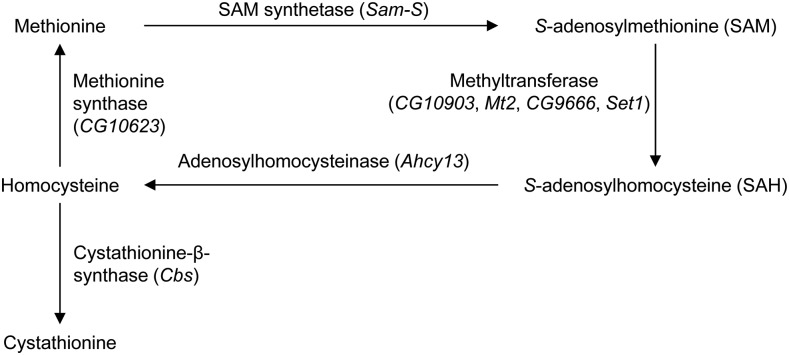
Methionine metabolism in *Drosophila*.

The metabolism of methionine is critical for the development of living organisms. *Sam-S* is an essential gene in *Drosophila* (Larsson and Rasmuson-Lestander 1998) and fungi (Gerke *et al.* 2012). Decreased level of SAM-S results in late flowering in rice *Oryza sativa* (Li *et al.* 2011). Depletion of AHCY or MS leads to lethality in mice (Miller *et al.* 1994; Swanson *et al.* 2001). Knockdown of *Cbs* leads to death in *Drosophila* (Kabil *et al.* 2011). Mice with CBS deficiency suffer from growth retardation and die within 5 weeks of birth (Watanabe *et al.* 1995). The metabolism of methionine is also important for cell proliferation. An *Escherichia coli Sam-S* mutant (*metK84*) shows slow growth and filamentation (Newman *et al.* 1998). Stable overexpression of AHCY induces cell death by apoptosis in human cells (Hermes *et al.* 2008). Reduction of CBS induces premature senescence in human endothelial cells (Albertini *et al.* 2012).

Given that SAM is the universal methyl donor, enzymes that control the levels of SAM play a critical role in determining the extent of histone methylation. RNA interference (RNAi) induced knockdown of *Sam-S* results in a reduction of global histone methylation in rice *Oryza sativa* (Li *et al.* 2011) and *Caenorhabditis elegans* (Towbin *et al.* 2012). AHCY deficiency in yeast inhibits histone methylation through increased SAH (Tehlivets *et al.* 2013). CBS-deficient mice have decreased asymmetric dimethylation of arginine 3 on histone H4 (H4R3me2a) relative to wild type in liver cells (Esse *et al.* 2014). Because histone methylation is related to gene transcription (Black and Whetstine 2011), it is possible that methionine metabolic enzymes regulate biological processes such as cell proliferation, development and the like through controlling genes involved in these processes, whose expression is affected by histone methylation (Teperino *et al.* 2010).

The levels of histone methylation are determined by the activities of histone methyltransferases and demethylases. Histone methylation affects gene expression, which in turn affects cell proliferation and development. H3K4 methylation is associated with active genes (Black and Whetstine 2011; Black *et al.* 2012). SET1 is a H3K4 methyltransferase conserved from yeast to human (Shilatifard 2012). Loss of SET1 leads to decreased H3K4 methylation and slow cell growth rate in yeast (Briggs *et al.* 2001). Reduced SET1 results in a decrease of global H3K4me2 and H3K4me3 levels and lethality in *Drosophila* (Ardehali *et al.* 2011; Mohan *et al.* 2011; Hallson *et al.* 2012). There are two orthologs of *Set1*, *Setd1a*, and *Setd1b*, in mammals (Shilatifard 2012). Both *Setd1a* and *Setd1b* are essential for development in mice, but only *Setd1a* is required for cell proliferation and H3K4 methylation in mouse embryonic stem cells (ESCs) (Bledau *et al.* 2014). To date, little imaginal discs (LID) and lysine-specific demethylase 2 (KDM2) are the only two *Drosophila* histone demethylases reported to target H3K4me3. *lid* and *Kdm2* genetically interact in *Drosophila* (Li *et al.* 2010). LID specifically removes H3K4me3 (Eissenberg *et al.* 2007; Lee *et al.* 2007; Secombe *et al.* 2007; Lloret-Llinares *et al.* 2008). LID is critical for *Drosophila* viability and development (Gildea *et al.* 2000; Li *et al.* 2010). Functions of KDM2 are controversial. KDM2 has been shown to influence H3K36me2 demethylation and H2A ubiquitylation in *Drosophila* S2 cells (Lagarou *et al.* 2008). KDM2 is also reported to target H3K4me3, but not H3K36me2, in *Drosophila* larvae (Kavi and Birchler 2009). Another group, however, determined that there is no change in H3K4me3 and H3K36me2 in wing imaginal discs from *Kdm2* mutants (Li *et al.* 2010). The differences between cells and larvae may result from different KDM2 complexes at different developmental stages or in different tissues (Zheng *et al.* 2014). Recently, KDM2 has been found to have weak effects on H3K4me3 and H3K36me1/2/3 in *Drosophila* larvae, but does not affect H3K4me1/2/3 and H3K36me1/2/3 in *Drosophila* S2 cells (Zheng *et al.* 2014). The contradictory results in S2 cells may be due to different *Kdm2* knockdown levels by using different dsRNA or differences between cells (Zheng *et al.* 2014). An initial report indicated that the strongest *Kdm2* mutant is lethal in flies (Lagarou *et al.* 2008). Two years later, another group, using a different set of alleles, reported that the strongest *Kdm2* mutant is semilethal (Li *et al.* 2010). Recent results from Ji’s group testing a number of alleles, including those tested in the first report, however, suggested that KDM2 is not required for *Drosophila* viability (Zheng *et al.* 2014). Analysis from Ji’s group demonstrated that the lethality observed in the *Kdm2* mutants is very likely due to second-site lethal mutations.

Taken together, research from multiple model organisms suggests that methionine metabolic enzymes, histone methyltransferases as well as demethylases are associated with histone methylation, cell proliferation and development. Whether methionine metabolic enzymes have similar effects on these biological processes in *Drosophila*, however, is understudied. The relationship among these enzymes in regulation of these processes is still not fully understood. Here, we have found that enzymes involved in methionine metabolism and histone demethylases play a role in development and cell proliferation in *Drosophila*. We also observed an interaction among these proteins in regulation of histone methylation. Together, our data provide insights into the connection between metabolism and epigenetics.

## Materials and Methods

### Cell culture

*Drosophila* Schneider cell line 2 (S2) cells were cultured at 27° in Schneider’s *Drosophila* medium (1x) with l-glutamine (Life Technologies), 10% heat-inactivated fetal bovine serum (Invitrogen), and 50 mg/ml gentamycin.

### Fly stocks

*Drosophila melanogaster* stocks were maintained and crosses were performed according to standard laboratory procedures. *Ser-Gal4* (#6791), *Act5C-Gal4* (#4414), *Engrailed-Gal4* (#30564), *UAS-GFP^RNAi^* (#9331), *UAS-Sam-S^RNAi-TRiP-1^* (#36306), *UAS-Sam-S^RNAi^*^-^*^TRiP-2^* (#29415), *UAS-Ahcy13^RNAi-TRiP^* (#51477), *UAS-Cbs^RNAi-TRiP^* (#36767), *UAS-CG10623^RNAi-TRiP^* (#51748), *UAS-CG10903^RNAi-TRiP^* (#57481), *UAS-Mt2^RNAi-TRiP^* (#38224), *UAS-Set1^RNAi-TRiP-1^* (#33704), *UAS-Set1^RNAi-TRiP-2^* (#38368), *UAS-lid^RNAi-TRiP^* (#28944), *UAS-Kdm2^RNAi-TRiP-1^* (#31360) and *UAS-Kdm2^RNAi-TRiP-2^* (#33699) were obtained from the Bloomington *Drosophila* Stock Center. *UAS-Cbs^RNAi-KK^* (#107325KK), *UAS-CG10623^RNAi-KK^* (#109718KK), *UAS-CG10903^RNAi-KK^* (#109610KK), *UAS-Mt2^RNAi-GD-1^* (#37815GD), *UAS-Mt2^RNAi-GD-2^* (#37816GD), *UAS-CG9666^RNAi-GD^* (#45658GD), *UAS-lid^RNAi-KK^* (#103830KK) and *UAS-Kdm2^RNAi-KK^* (#109295KK) were obtained from the Vienna *Drosophila* Research Center. *hsFLP;Act5C > CD2 > GAL4,UAS-EGFP* was kindly provided by Dr. Dirk Bohmann (University of Rochester Medical Center).

### dsRNA production

The protocols to generate constructs containing targeting sequences in pCRII-Topo vector and to produce dsRNA are described previously (Pile *et al.* 2002). The sequences in the pCRII-Topo vector were generated using specific primer pairs (Supporting Information, Table S1). *Set1* primers were found on DRSC FlyPrimerBank (http://www.flyrnai.org/FlyPrimerBank) (Hu *et al.* 2013). Primers for *lid* were found on Genome RNAi (http://www.genomernai.org) (Schmidt *et al.* 2013). The rest of the primers were taken from *Drosophila* RNAi Library 1.0 and *Drosophila* RNAi Library 2.0 on Open Biosystems. dsRNA against green fluorescent protein (GFP) prepared from a PCR product was used as a control. GFP template DNA (from Dr. Russell L. Finley, Jr.) was amplified using a T7-containing primer pair (Table S1).

### RNA interference

The RNA interference (RNAi) procedure is described previously (Pile *et al.* 2002). In brief, 3 × 10^6^ cells with 4 ml Schneider’s *Drosophila* medium were plated in a 60-mm-diameter dish. After 3 hr, Schneider’s *Drosophila* medium was removed and replaced with 2 ml serum-free medium. 50 μg dsRNA was added into the dish and mixed by swirling. After 30 min, 4 ml Schneider’s *Drosophila* medium was added. Cells were assayed 4 days following addition of dsRNA. dsRNA against GFP was used as the control. Real-time quantitative reverse transcription PCR (qRT-PCR) analysis was routinely carried out for both single- and double-RNAi-treated cells to verify efficient knockdown of targets.

### Real-time quantitative reverse transcription PCR assay

Total RNA was extracted from whole flies or wing imaginal discs using Trizol (Invitrogen) and purified using the RNeasy Mini Kit (Qiagen). Total RNA was extracted from RNAi-treated cells using the RNeasy Mini Kit (Qiagen). cDNA was generated from total RNA using the ImProm-II Reverse Transcription System (Promega) with random hexamers. The cDNA was used as a template in a real-time quantitative PCR assay. The analysis was performed using ABsolute Blue SYBR Green ROX master mix (Fisher Scientific) and carried out in a Stratagene Mx3005P real-time thermocycler. *Taf1* was used to normalize RNA levels. The mRNA levels were quantified using real-time quantitative PCR with specific primers for each gene (Table S2). Primers for *Mt2* were found on DRSC FlyPrimerBank (http://www.flyrnai.org/FlyPrimerBank) (Hu *et al.* 2013). Primers for *Set1* were taken from a previously published report (Ardehali *et al.* 2011). The gene expression changes are represented as the mean (± SEM) of the fold changes observed in the fly lines or RNAi-treated cells. In whole flies, fold differences were calculated by relative comparison of flies *Act5C-Gal4/UAS-GOI (gene of interest)^RNAi^* to *Act5C-Gal4/UAS-GFP^RNAi^* flies. In lines where ubiquitous knockdown was lethal, fold differences were calculated by relative comparison of *Ser-Gal4/UAS-GOI^RNAi^* wing imaginal discs to *Ser-Gal4/UAS-GFP^RNAi^* wing imaginal discs. In cells, fold differences were calculated by relative comparison of GOI RNAi cells to GFP RNAi cells. This experiment utilized a minimum of three sets of RNA for each cell type or fly line.

### Fly viability

Viability of flies with ubiquitous knockdown of each gene was measured by crossing *Act5C-Gal4/CyO* flies to RNAi lines of each gene of interest. The percent viability was calculated by dividing the number of *Act5C-Gal4;RNAi* progeny by the number of *CyO;RNAi* progeny. Three biological replicates were performed.

### Fly wing phenotype

The effect of knockdown of each gene in the wing imaginal discs of knockdown fly lines was examined by crossing *Ser-Gal4* flies to RNAi lines of each gene of interest. Wings of *Ser-Gal4;RNAi* progeny were scored for shape, vein defects and curvature. Three biological replicates were conducted.

### Clonal analysis

*hsFLP;Act5C > CD2 > GAL4,UAS-EGFP* flies were crossed to *mCherry^RNAi-TRiP^* or to the RNAi lines of each gene of interest to generate random GFP-positive clones. Embryos (0–4 hr) were collected and heat-shocked for 2 hr at 37°, 48–52 hr after egg laying (AEL). Wing discs from wandering third instar larvae were dissected approximately 96 hr AEL and immunostained with monoclonal antibody against GFP.

### Immunostaining

For clonal analysis, wing discs from wandering third instar larvae were dissected in 1X PBS. Roughly 70 wing discs per cross (obtained from three biological replicates) were fixed in 4% formaldehyde in 1X PBS and stained as previously described (Swaminathan and Pile 2010). Antibody against GFP (1:1000; Abcam, ab1218) followed by sheep anti-mouse Alexa 488 (1:2000; Life Technologies, A11001) was used for staining. The GFP-positive clone pixel count was quantified using Photoshop CS. For H3K4me2 staining, wing discs from wandering third instar larvae were dissected in 1X PBS. Roughly 60 wing discs per cross (obtained from three biological replicates) were fixed in 4% formaldehyde in 1X PBS and stained as previously described (Swaminathan and Pile 2010). Antibody against dimethyl-histone H3 (Lys4) (1:1000; Millipore 07-030) followed by donkey anti-rabbit Alexa 594 (1:2000; Life Technologies, A11001) was used for staining. Discs for both clonal analysis and H3K4me2 staining were mounted in Vectashield medium plus DAPI (Vector Laboratories; H-1200). Visualization and imaging (200X) were done using a Zeiss Axioscope 2 fitted with an Axiophot photography system.

### Imaging

Wing images (63X and 115X) and adult fly images (30X) were taken with an Olympus DP72 camera coupled to an Olympus SZX16 microscope.

### S2 cell proliferation assay

Four days after RNAi treatment, RNAi-treated cells were stained with Trypan Blue. The number of cells per milliliter in each sample was calculated by following hemacytometer standard protocol. The experiments were repeated with three biological trials.

### Western blotting analysis

Western blot analysis of whole cell protein extract was performed in accordance with standard protocols as described previously (Pile *et al.* 2002). Whole cell protein extracts (12 μg) were separated on a 15% sodium dodecyl sulfate (SDS)-polyacrylamide gel and transferred to a polyvinylidene difluoride (PVDF) membrane (Pall). Membranes were probed with various rabbit primary antibodies followed by incubation with donkey anti-rabbit HRP-conjugated IgG (1:3000; GE Healthcare) secondary antibody. The antibody signals were detected using the clarity western ECL substrate (Bio-Rad) for H3K4me2 and H3K4me3 or ECL prime western blot detection system (GE Healthcare) for H3K9me2 and H4. Primary antibodies included: H3K4me2 (1:5000; Millipore), H3K4me3 (1:2500; Active Motif), H3K9me2 (1:500; Millipore) and H4 (1:15,000; Abcam) as a loading control. At least three biological replicates were performed.

### Chromatin immunoprecipitation and real-time quantitative PCR

To prepare chromatin, 4 × 10^7^ cells were subjected to cross-linking with 1% formaldehyde for 10 min and quenched with 125 mM glycine at room temperature. Cells were then washed three times with 1X PBS at 4° for 5 min and were resuspended in 15 ml resuspension buffer [10 mM Tris (pH 8), 10 mM KCl, 3 mM CaCl_2_, 0.34 M Sucrose, 1 mM DTT, 0.1% Triton X-100, 0.2 mM EGTA, 1.5 Roche complete protease inhibitor tablet]. The resuspended cells were incubated on ice for 15 min and then homogenized by a dounce homogenizer using a loose pestle 10 times and a tight pestle 15 times followed by low speed centrifugation at 4° for 10 min. The loose pellet was resuspended in 200 μl 10X MNase digest buffer [15 mM Tris (pH 8), 60 mM KCl, 15 mM NaCl, 1 mM CaCl_2_, 0.25 M sucrose, 1 mM DTT] and subjected to MNase digestion using 20 units of MNase for 30 min at room temperature; 10 mM EDTA was added to stop the reaction. Samples were diluted with 950 μl Ten140 buffer [140 mM NaCl, 10 mM Tris (pH 7.6), 2 mM EDTA]. The samples were then subjected to sonication for seven times of 30 sec pulses with 1-min intervals at 20% amplitude using an Ultrasonic dismembrator Model 500, Fisher Scientific) sonicator. Sonicated samples were centrifuged for 15 min at 4° and the chromatin was in the supernatant.

To prepare input DNA, 18.75 μg sonicated chromatin diluted to a final volume of 250 μl Ten140 buffer was used. Chromatin was treated with 0.05 μg/μl RNase A at 37° for 15 min and then incubated overnight at 65° after adding 200 mM NaCl. Samples were then treated with 0.04 μM Proteinase K (Fisher Scientific), 10 μM EDTA, 20 μM Tris (pH 8.0) at 45° for 1.5 hr and subjected to phenol chloroform extraction and ethanol precipitation. Precipitated DNA was resuspended in 25 μl 0.1 mM Tris (pH 8.0).

For immunoprecipitation, 75 μg prepared chromatin was diluted to a final volume of 500 μl Ten140 buffer. IgG was used as a nonspecific control and H3 acted as a loading control. Chromatin was incubated overnight with 2.5 μl IgG, 3 μl H3K4me3 (Active Motif), or 4 μl H3 (Abcam) antibody on a nutator at 4°. Samples were then mixed with 30 μl anti-IgG beads (Protein A agarose, Pierce), which were prewashed six times with lysis buffer [50 mM Tris (pH 7.6), 280 mM NaCl, 2 mM EDTA, 0.3% SDS]. The samples with beads were placed on a nutator at 4° for 4 hr. Anti-IgG beads were then washed with 1 ml lysis buffer for 5 min, 1 ml IP 1 buffer [25 mM Tris (pH 7.6), 500 mM NaCl, 1 mM EDTA, 0.1% sodium deoxycholate, 1% Triton X-100] for 10 min and 1 ml IP 2 buffer [10 mM Tris (pH 7.6), 250 mM LiCl, 1 mM EDTA, 0.5% sodium deoxycholate, 0.5% Triton X-100] for 5 min at 4°. The beads were then rinsed with 1 ml Tris-EDTA (pH 8.0) and incubated with 500 μl elution buffer (1% SDS, 0.1 M NaHCO_3_) at 65° for 1 hr. Eluted samples were subject to reverse cross-linking in the same way as described above for input preparation. Precipitated DNA was resuspended in 50 μl 0.1 mM Tris (pH 8.0).

Input DNA (diluted 1:100) and immunoprecipitated samples (diluted 1:4) were used as the template in a real-time quantitative PCR assay. The analysis was performed using ABsolute Blue SYBR Green ROX master mix (Fisher Scientific) and carried out in a Stratagene Mx3005P real-time thermocycler. Primers are listed in Table S3.

### Statistical analyses

All significance values were calculated by the unpaired two sample Student‘s *t*-test from GraphPad Software (http://www.graphpad.com/quickcalcs/ttest1/).

### Data availability

The primers used to generate dsRNA can be found in Table S1. The primers used for gene expression and ChIP-qPCR analysis can be found in Table S2 and Table S3, respectively.

## Results

### Functions of genes involved in methionine metabolism and histone demethylase genes in *Drosophila* viability and wing morphology

To address the role of enzymes in methionine metabolism and histone demethylases in regulating developmental processes, we utilized RNAi to ubiquitously knock down the genes of interest through the GAL4-UAS system (Duffy 2002; Lee and Carthew 2003). To date, according to the information on Flybase (http://flybase.org) (St Pierre *et al.* 2014), *Sam-S* is the only known SAM synthetase gene (Larsson and Rasmuson-Lestander 1994), *Ahcy13* is the major adenosylhomocysteinase gene (Caggese *et al.* 1997), and *Cbs* is the only known cystathionine-β-synthase gene in *Drosophila*. *CG10623* may encode a putative methionine synthase. CG10903 is predicted to have SAM-dependent methyltransferase activity and is reported to positively affect cell proliferation in *Drosophila* neural stem cells (Neumuller *et al.* 2011). CG9666 is a predicted N6-adenine specific DNA methyltransferase based on sequence and structural analysis. *Mt2* is a candidate CpG DNA methyltransferase gene in *Drosophila*, but this type of DNA methyltransferase activity is controversial (Tang *et al.* 2003; Raddatz *et al.* 2013; Dunwell and Pfeifer 2014). In addition, MT2 is reported to have tRNA methyltransferase activity (Schaefer *et al.* 2010). We chose these seven genes, as well as a known histone methyltransferase gene *Set1* and two histone demethylase genes, *lid* and *Kdm2*, to investigate their role in development.

To knock down the genes of interest, we crossed *UAS-RNAi* fly lines to the *Act5C-Gal4* driver line. Progeny will ubiquitously express dsRNA recognizing the target, so the expression of the gene will be knocked down in all tissues. We refer to these offspring as deficient flies. To rule out the possibility that phenotypes observed in the RNAi knockdown fly lines are the result of an off-target effect, we utilized more than one *UAS-RNAi* line for each gene whenever possible. We utilized multiple targeting lines for all genes with the exception of *Ahcy13* and *CG9666*. The efficiency of knockdown was validated by qRT-PCR analysis (Figure S1). Individual ubiquitous knockdown of methionine metabolic genes *Sam-S*, *Ahcy13*, *Cbs*, but not *CG10623*, resulted in lethality (Table 1). These data are consistent with previous studies demonstrating that *Sam-S* and *Cbs* are essential genes (Larsson and Rasmuson-Lestander 1998; Kabil *et al.* 2011) and indicate that the majority of enzymes in the methionine pathway are critical for fly viability. For three genes annotated to have methyltransferase activity, deficiency of *CG10903*, but not *Mt2* and *CG9666*, affected viability (Table 1). *Mt2* was previously demonstrated to be nonessential for viability (Lin *et al.* 2005). Reduction of histone methyltransferase Set1 or demethylase LID impaired viability in our hands (Table 1), which is consistent with previously published work (Gildea *et al.* 2000; Hallson *et al.* 2012). The effect of KDM2 in *Drosophila* viability is controversial (Lagarou *et al.* 2008; Li *et al.* 2010; Zheng *et al.* 2014), but our results support the idea that *Kdm2* is not essential (Table 1). Similar results were obtained from the different RNAi fly lines targeting the same gene. Thus, it is very likely that the observed lethality is due to the reduction of the specific gene tested, and not the result of an off-target effect. The viability data demonstrate that some but not all tested methyltransferases and demethylases are essential. Although the genes selected have been shown or predicted to be a methyltransferase or demethylase, some may be redundant with other enzymes or may affect specific methylation or demethylation reactions that are not essential. These viability tests, consistent with published work, indicate that enzymes involved in methionine metabolism, histone methylation and demethylation are necessary for development in *Drosophila*.

**Table 1:** Ubiquitous knockdown of *Sam-S*, *Ahcy13*, *Cbs*, *CG10903*, *Set1*, or *lid* results in a loss of viability

		♂			♀		
			Flies Scored			Flies Scored	
Gene	Stock Name (*UAS-GOI*)	% Viable	*Act5C-Gal4/UAS-GOI*	*CyO/UAS-GOI*	% Viable	*Act5C-Gal4/UAS-GOI*	*CyO/UAS-GOI*
*Sam-S*	*Sam-S^RNAi-TRiP-1^*	0	0	180	0	0	222
	*Sam-S^RNAi-TRiP-2^*	0	0	178	0	0	190
*Ahcy13*	*Ahcy13^RNAi-TRiP^*	0	0	137	0	0	188
*Cbs*	*Cbs^RNAi-KK^*	0	0	146	0	0	183
	*Cbs^RNAi-TRiP^*	0	0	146	0	0	183
*CG10623*	*CG10623^RNAi-KK^*	98.7 ± 24.1	231	273	98 ± 12.5	229	249
	*CG10623^RNAi-TRiP^*	96 ± 21.5	195	203	99 ± 7.2	217	222
*CG10903*	*CG10903^RNAi-KK^*	0	0	144	0	0	187
	*CG10903^RNAi-TRiP^*	0	0	113	0	0	157
*Mt2*	*Mt2^RNAi-GD-2^*	93.3 ± 11.6	144	159	123 ± 20	195	163
	*Mt2^RNAi-TRiP^*	102.3 ± 11.3	187	184	99.7 ± 3	187	188
*CG9666*	*CG9666^RNAi-GD^*	139 ± 10.4	146	110	85.7 ± 7.6	125	154
*Set1*	*Set1^RNAi-TRiP-1^*	0	0	194	0	0	192
	*Set1^RNAi-TRiP-2^*	0	0	133	0	0	169
*lid*	*lid^RNAi-KK^*	0	0	122	0	0	237
	*lid^RNAi-TRiP^*	0	0	231	0	0	334
*Kdm2*	*Kdm2^RNAi-KK^*	98 ± 19.7	138	148	90.4 ± 13	147	167
	*Kdm2^RNAi-TRiP-1^*	98.3 ± 10.2	137	143	106.3 ± 2.4	176	165
	*Kdm2^RNAi-TRiP-2^*	103 ± 29.4	191	185	99.1 ± 8.9	180	182

The percent viability is calculated by dividing the number of *Act5C-Gal4/UAS-GOI* progeny by the number of *CyO/UAS-GOI* progeny. Standard error of the mean is indicated. Three trials were performed. GOI, gene of interest; TRiP, Transgenic RNAi Project at Harvard Medical School; KK, ΦC31 Transgenic RNAi Library; GD, P-element Transgenic RNAi Library.

**Figure 2:**
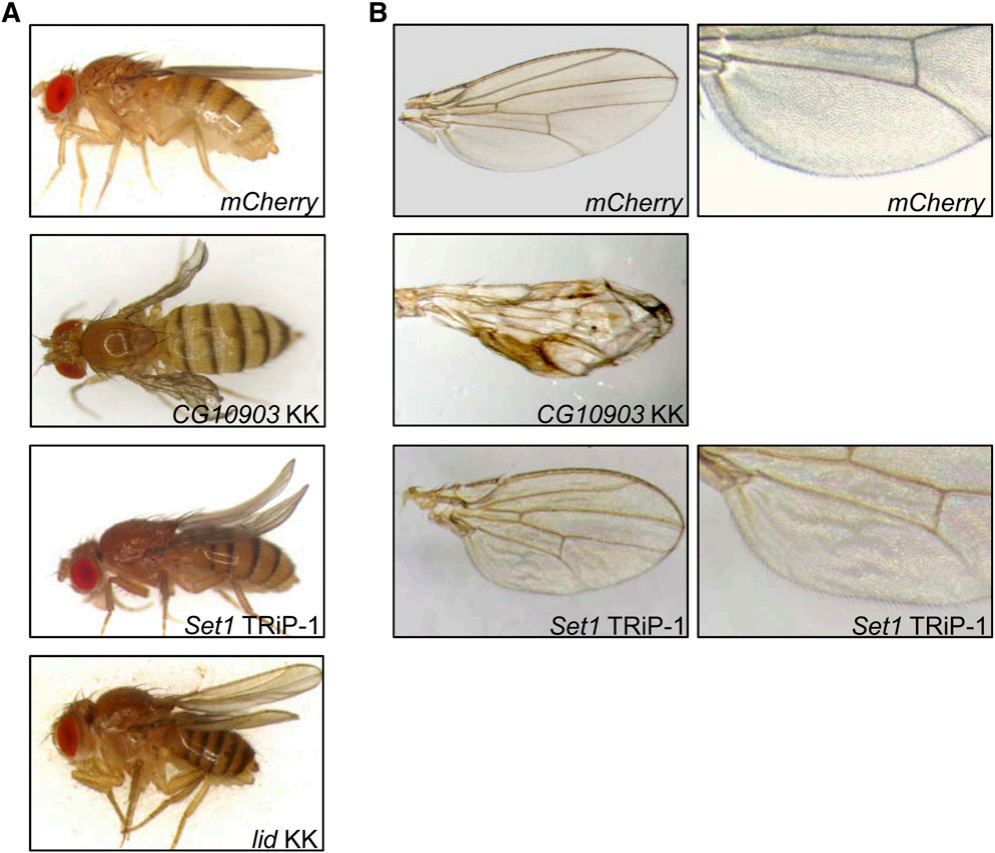
*CG10903*, *Set1*, and *lid* affect wing morphology. Micrographs of flies (A) or wings (B) carrying the *Ser-Gal4* driver and the indicated *UAS-RNAi* constructs. *mCherry*: control flies expressing *mCherry* dsRNA. Flies with knockdown of *Sam-S*, *Ahcy13*, *Cbs*, *CG10623*, *Mt2*, *CG9666* and *Kdm2* had straight wings, similar to the control. For each knockdown sample, at least 174 flies from three biological replicates were scored. All progeny in the same knockdown sample showed the same phenotype.

The lethality caused by ubiquitous reduction of methionine metabolic enzymes and histone modifiers led us to further investigate their role in development using a conditional knockdown system. Wing tissue is nonessential and has been used by us and others to explore developmental functions of individual factors. We conditionally knocked down each tested gene in wing imaginal disc cells by activating expression of specific dsRNA targeting sequences with the wing specific driver *Ser-Gal4*. Knockdown of *CG10903* in wing imaginal disc cells using the *UAS-CG10903^RNAi-KK^* line resulted in severely wrinkled, blistered adult wings in all progeny (Figure 2). The use of the *UAS-CG10903^RNAi-TRiP^* line led to lethality in the pupal stage of development. The different results are possibly due to the measured differences in RNAi efficiency of the dsRNA constructs utilized (Figure S1). While we do not have a definitive explanation for the lethality caused by reduced CG10903 in wing tissue, it may be related to a “molting checkpoint” (Cherbas *et al.* 2003). As proposed in Cherbas *et al.* (2003), due to the presence of a molting checkpoint, tissue-specific reduced expression of a particular gene may block development of the entire animal, leading to lethality. Reduction of SET1 in wing precursor cells led to a curved rather than straight adult wing in all offspring (Figure 2A and Figure S2A) and a ruffled wing between veins L5 and L6 (Figure 2B and Figure S2B). In accord with previous work indicating that *lid* can genetically interact with *Notch* or *snr1* to affect wing vein development (Moshkin *et al.* 2009; Curtis *et al.* 2011), we found decreased LID resulted in a curved wing in all progeny (Figure 2A and Figure S2A). The multiple RNAi lines tested each yielded very similar results indicating that the wing defects are specific for SET1 or LID and are not due to off target effects. All flies with reduced SAM-S, AHCY13, CBS, CG10623, MT2, CG9666 or KDM2 showed normal adult wings (data not shown). Taken together, these observations indicate that while the methionine metabolic pathway is not critical for wing development, direct regulation of histone methylation and demethylation plays an important role in the development of normal wing morphology in *Drosophila*.

### Cell proliferation is modulated by altering the levels of enzymes involved in methionine metabolism and a histone demethylase

**Figure 3:**
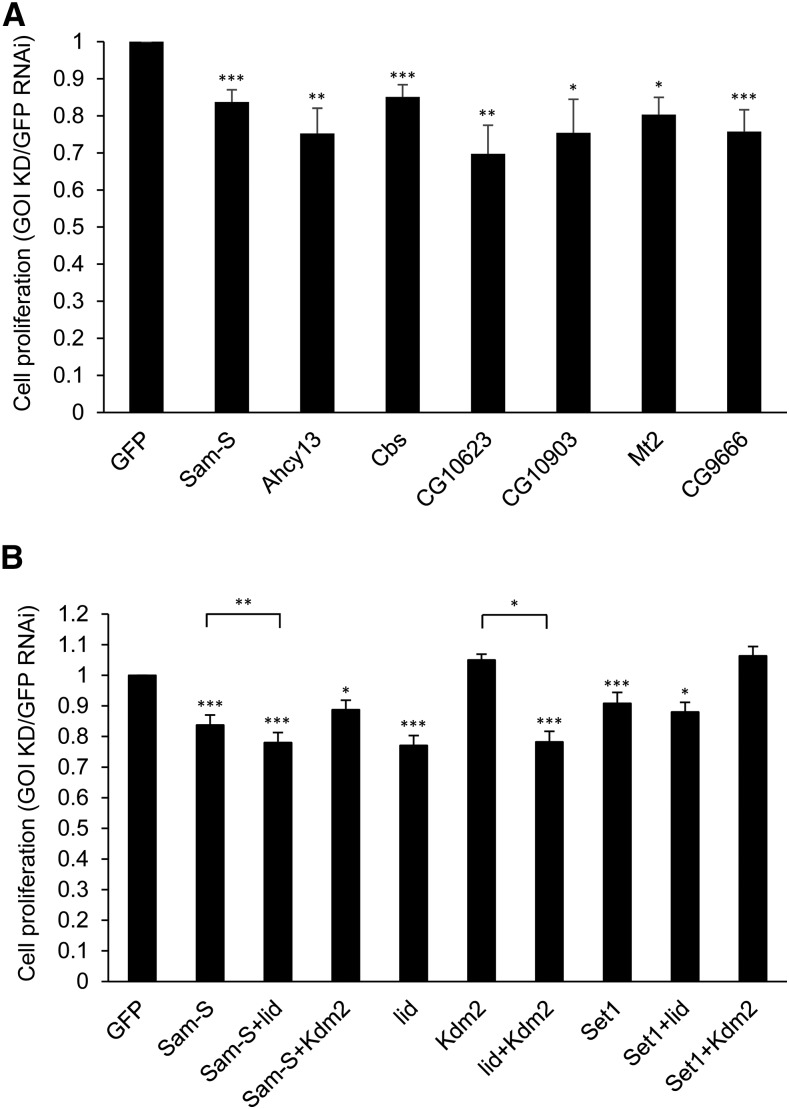
Cell proliferation in S2 cells is affected by methionine metabolic enzymes and histone methyltransferases and demethylases. (A, B) Quantification of cell density by cell counts from RNAi-treated cells. Results are the average of three biological replicates. Error bars represent standard error of the mean. Statistically significant results comparing the individual knockdown samples to the GFP RNAi control are indicated on knockdown samples. *P*-values were also calculated between the double knockdown samples and each single knockdown sample for the two tested genes, *e.g.*, *Sam-S*+*lid* to *Sam-S* and *Sam-S*+*lid* to *lid*. Statistically significant results are indicated with bars. (*) *P* < 0.05, (**) *P* < 0.01, (***) *P*< 0.001. GFP: control cells treated with dsRNA against GFP. GOI KD: cells treated with dsRNA against the gene(s) of interest.

Abnormal wing development has been found to occur when normal cell proliferation pathways are mutated (Herranz and Milan 2008). Additionally, altered levels of enzymes controlling methionine metabolism can affect cell proliferation in human cells (Hermes *et al.* 2008; Albertini *et al.* 2012). For these reasons, we decided to determine whether methionine metabolic enzymes, histone methyltransferases and demethylases contribute to regulation of cell proliferation in *Drosophila*. We first checked for defects in cell proliferation by measuring cell number in *Drosophila* S2 cells upon RNAi-mediated depletion of genes of interest. For these experiments, the level of gene knocked down was determined by qRT-PCR analysis (Figure S3). Compared to control cells treated with GFP dsRNA, individual knockdown of all tested genes, except *Kdm2*, led to lower cell counts with a range of 10%–30% decrease (Figure 3).

To further investigate the relationship among these enzymes in affecting cell proliferation, we measured cell counts in S2 cells having different combinations of knockdown factors. Because we observed a connection between SAM-S and histone demethylases as well as between SET1 and histone demethylases in regulation of histone methylation (described in detail below), we focused on the same combinations to determine their role in cell growth. Double knockdown cells did not show an additive effect on cell proliferation relative to the single knockdown cells (Figure 3B). The double knockdown cells showed cell numbers comparable to the single knockdown cells that had the larger effect between two tested genes (Figure 3B). These results imply that methionine metabolism, histone methylation and demethylation probably influence the same pathway(s) to regulate cell proliferation in this cell type.

**Figure 4:**
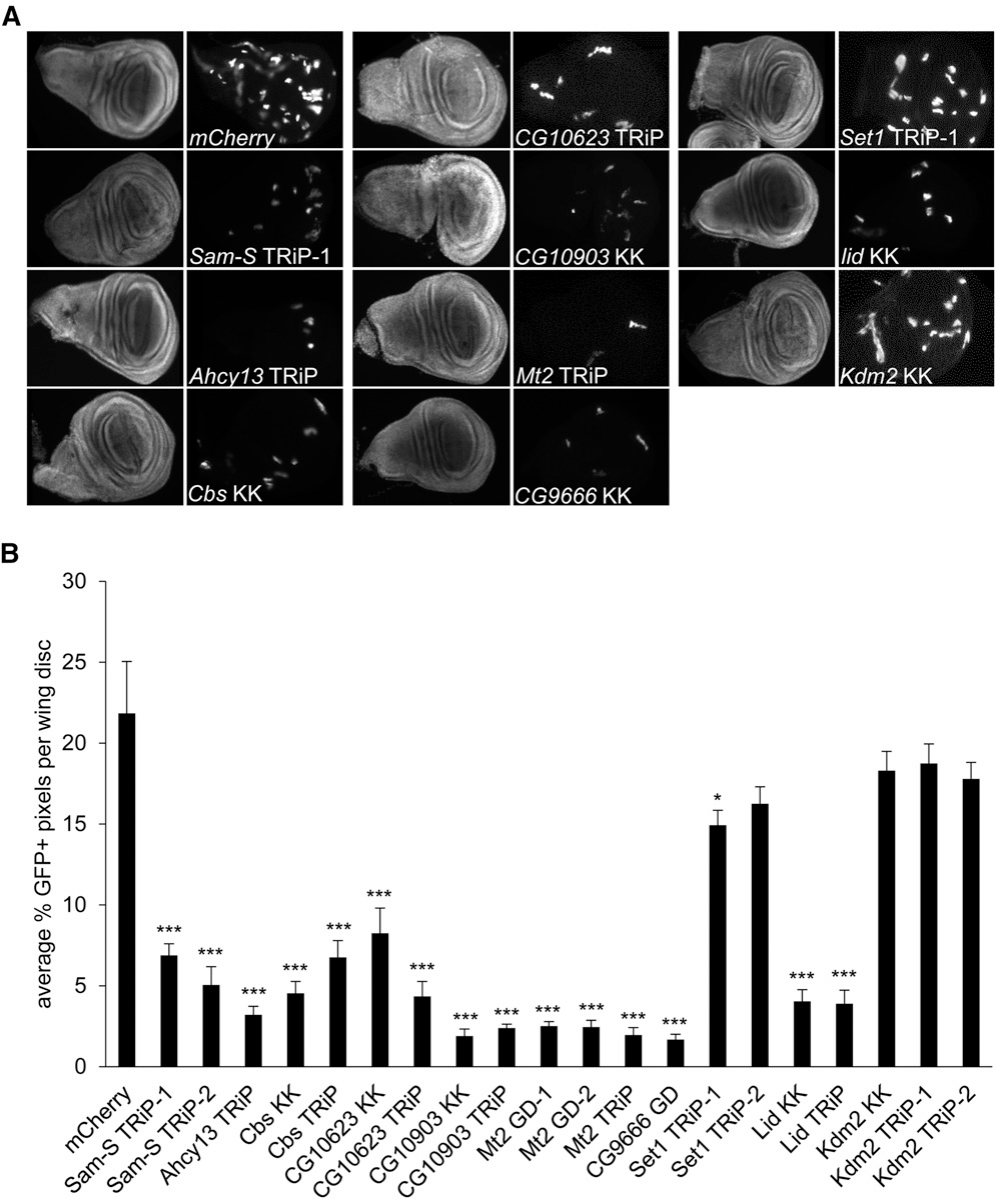
Cell proliferation in wing imaginal discs is affected by methionine metabolic enzymes and histone methyltransferases and demethylases. (A) *mCherry* RNAi control and knockdown wing disc clones were generated using the flip-out GAL4 system and immunostained with antibody to GFP. GFP signal is shown in the right panel of paired images for each fly line. DAPI staining is in the left panel. (B) Quantification of GFP signal in wing imaginal discs. Results are the average of GFP positive pixel counts from three biological replicates with at least 70 wing imaginal discs in total for each knockdown sample. Error bars represent standard error of the mean. *P*-values were calculated by comparing the GFP positive pixel count measured in the individual knockdown fly to the GFP positive pixel count in the *mCherry* RNAi control. Statistically significant results are indicated on knockdown samples. (*) *P* < 0.05, (***) *P* < 0.001. *mCherry*: control fly carrying dsRNA against *mCherry*.

All tested genes, except *Kdm2*, affected cell proliferation in S2 cells, which prompted us to analyze the role of these genes in cell growth during fly development. To address this question, we performed clonal analysis in *Drosophil*a wing imaginal discs. We utilized the heat shock flip-out system to randomly generate clones expressing GFP in RNAi knockdown cells. Reduction of all tested enzymes, except KDM2, resulted in small GFP positive clones that were fewer in number relative to the *mCherry* RNAi control (Figure 4). The defects of cell proliferation in wing imaginal discs are specific for the targeted genes as they could be confirmed using a second RNAi line, except *Ahcy13* and *CG9666* as mentioned (Figure 4B and Figure S4). Collectively, data from cultured cells and developing flies demonstrate that the methionine pathway, histone methylation and demethylation have a critical role in the regulation of cell proliferation.

### Disruption of methionine metabolism affects histone methylation

**Figure 5:**
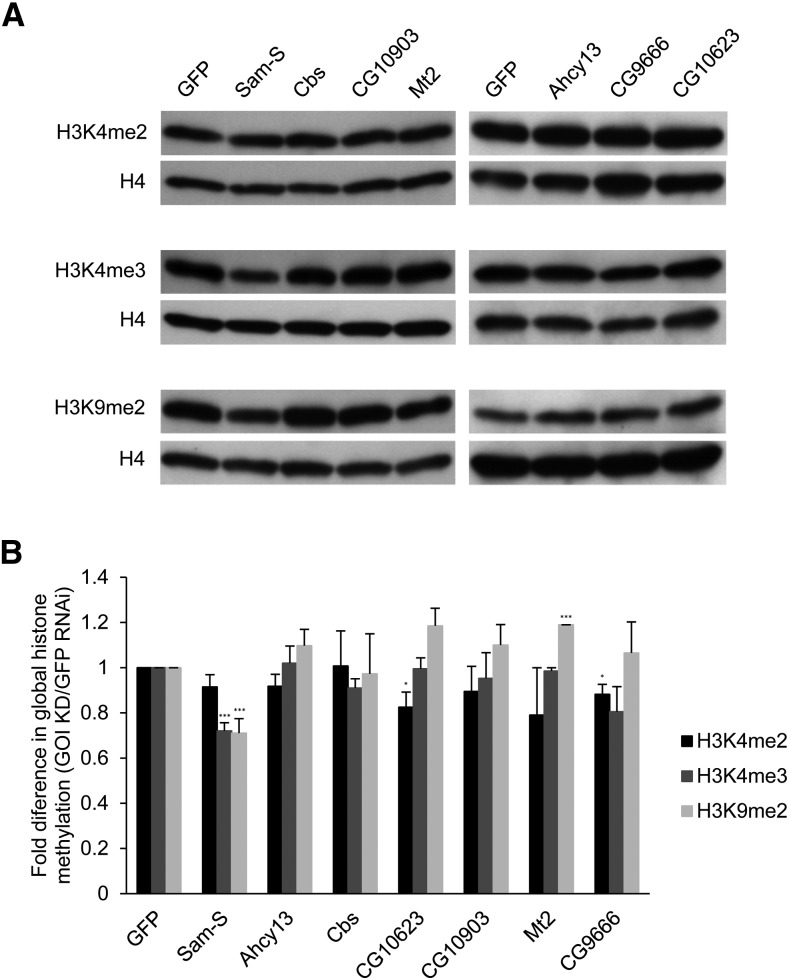
Global histone methylation levels in S2 cells are regulated by enzymes involved in methionine metabolism. (A) Whole cell extracts from RNAi-treated cells were subjected to western blot analysis for H3K4me2, H3K4me3, H3K9me2 or H4. (B) Western blots as shown in A were repeated with protein extracts prepared from at least three independent cultures and the results were quantified after normalization to histone H4. Error bars represent standard error of the mean. *P*-values were calculated between the individual knockdown sample and the GFP RNAi control. Statistically significant results are indicated. (*) *P* < 0.05, (***) *P* < 0.001. GFP: control cells treated with dsRNA against GFP. GOI KD: cells treated with dsRNA against the gene of interest.

Given that methionine metabolism generates the major methyl donor SAM, we wanted to characterize the functions of genes involved in methionine metabolism in the possible modulation of histone methylation levels. It is known that H3K4 methylation is associated with active transcription, whereas H3K9 methylation is associated with repressive transcription (Black *et al.* 2012). We performed western blot analysis of whole cell protein extracts from *Drosophila* S2 cells with RNAi-mediated depletion of genes of interest. The blots were probed with antibodies specific for H3K4me2, H3K4me3, H3K9me2 as well as histone H4 as the loading control (Figure 5A). Global histone methylation levels normalized with histone H4 in each condition were quantified (Figure 5B). Knockdown of *Sam-S* resulted in decreased global H3K4me3 and H3K9me2 levels. Reduction of *Mt2* led to increased global H3K9me2 levels. Global H3K4me2 levels were reduced upon decreased expression of *CG10623* or *CG9666* relative to control GFP dsRNA-treated cells. The decrease in the level of H3K4me2 in S2 cells, while small, was reproducible. To further analyze the possible function of CG10623 or CG9666, we looked at global H3K4me2 levels during fly development. We crossed the *UAS-RNAi* fly lines to the *Engrailed-Gal4* driver line. Targeted genes are knocked down in the posterior region of wing imaginal discs in the progeny. No obvious changes of H3K4me2 levels were found between posterior and anterior compartments of wing imaginal discs when CG10623 or CG9666 was reduced (Figure S5). The impact of these enzymes on downstream processes in S2 cells or on H3K4me2 in other cell types remains open for further study.

**Figure 6:**
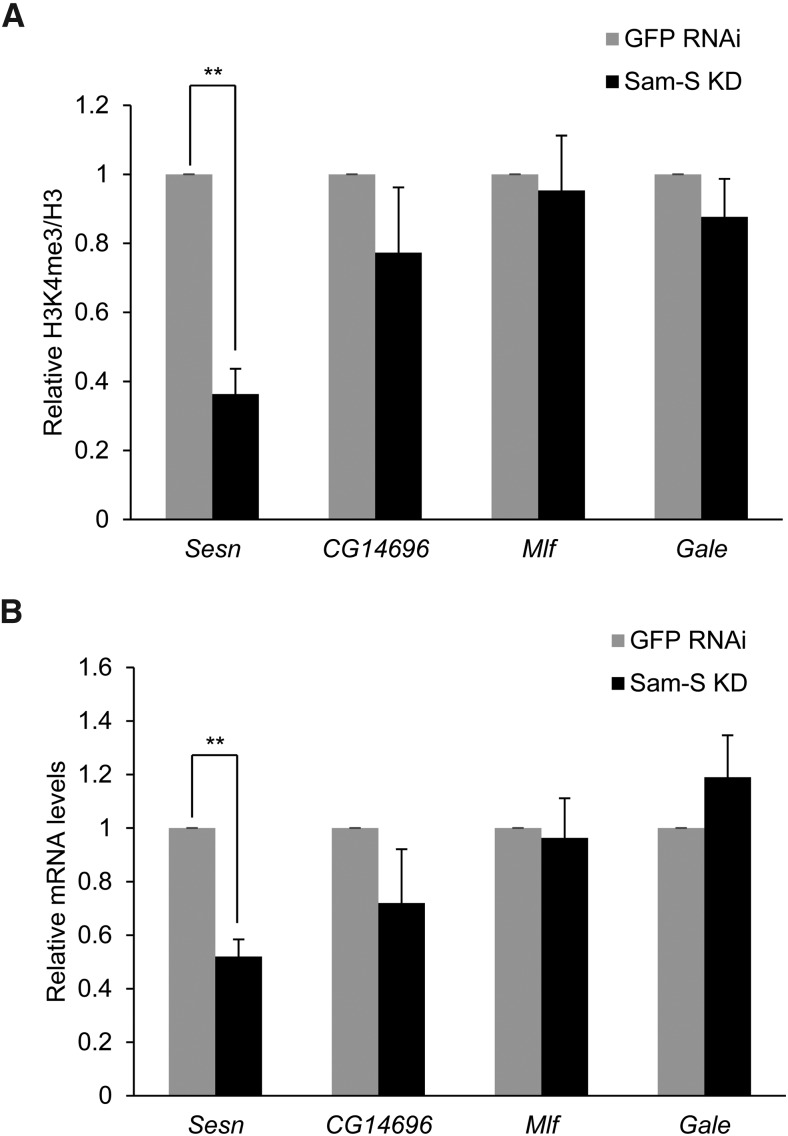
Knockdown of *Sam-S* affects gene specific H3K4me3 levels and gene expression. (A) ChIP-qPCR analysis of H3K4me3 levels at selected genes in GFP RNAi control and *Sam-S* deficient cells. Real-time quantitative PCR analysis was performed using chromatin prepared from RNAi-treated S2 cells immunoprecipitated with antibody to H3K4me3 or H3. H3K4me3 levels were normalized to histone H3 at specific genes. Relative H3K4me3 signals were calculated by dividing normalized H3K4me3 levels in *Sam-S* deficient cells by the levels in GFP RNAi control. Results are the average of three biological replicates. Error bars represent standard error of the mean. Statistically significant results are indicated. (B) Expression of selected genes in GFP RNAi control and *Sam-S* deficient cells. *Taf1* was used to normalize expression levels. The relative mRNA was calculated by dividing normalized gene expression levels in *Sam-S* deficient cells by the levels in GFP RNAi control. Results are the average of three biological replicates. Error bars represent standard error of the mean. Statistically significant results are indicated. (**) *P* < 0.01.

The finding that *Sam-S* affects global histone methylation led us to further investigate the activity of this key enzyme in regulation of gene specific histone methylation marks. Because H3K4me3 is an important histone mark associated with active genes (Black and Whetstine 2011; Black *et al.* 2012), we focused on this histone modification for further study. We selected four genes, *Sesn*, *CG14696*, *Mlf* and *Gale*, based on the published ChIP-seq analysis of H3K4me3 levels in S2 cells (Gan *et al.* 2010). *Sesn* and *Mlf* are implicated in cell proliferation (Jasper *et al.* 2002; Lee *et al.* 2010). *Gale* encodes UDP-galactose 4’-epimerase, which is involved in the galactose metabolic process (Sanders *et al.* 2010). The function of *CG14696* is unknown. Gan *et al.* (2010) determined that the promoter regions of *Mlf* and *Gale* have the highest H3K4me3 levels in the whole genome, *sesn* has middle H3K4me3 levels, *CG14696* has the lowest, yet still observable, H3K4me3 levels of these four genes. To examine if SAM-S affects the enrichment of H3K4me3 at these four genes, we performed ChIP-qPCR analysis using chromatin prepared from GFP RNAi control and *Sam-S* deficient S2 cells and immunoprecipitated with antibody to IgG, H3K4me3 or H3 (Figure S6). IgG was used as a nonspecific control. Histone H3 signal was used to normalize H3K4me3 levels. Typically, a more than 100-fold enrichment of H3K4me3 or H3 compared to IgG was observed at all regions sampled (Figure S6). Knockdown of *Sam-S* led to a significant decrease of H3K4me3 levels at *Sesn*, a nonsignificant decrease at *CG14696* and *Gale*, and no change at *Mlf* (Figure 6A). Given that H3K4me3 is associated with active genes (Black and Whetstine 2011; Black *et al.* 2012), we predicted that decreased H3K4me3 would result in a decline in gene expression. To test this hypothesis, we measured expression of these four genes in GFP RNAi control and *Sam-S* deficient S2 cells by qRT-PCR analysis. A significant decrease of *Sesn* expression was observed when SAM-S was reduced, while expression of the other three genes was not significantly changed (Figure 6B). Taken together, these data reveal that SAM-S regulates global and gene specific histone methylation, which is associated with gene expression.

### Decreased global H3K4me3 levels resulting from reduced SAM-S or SET1 are restored to near control levels upon *lid* knockdown

**Figure 7:**
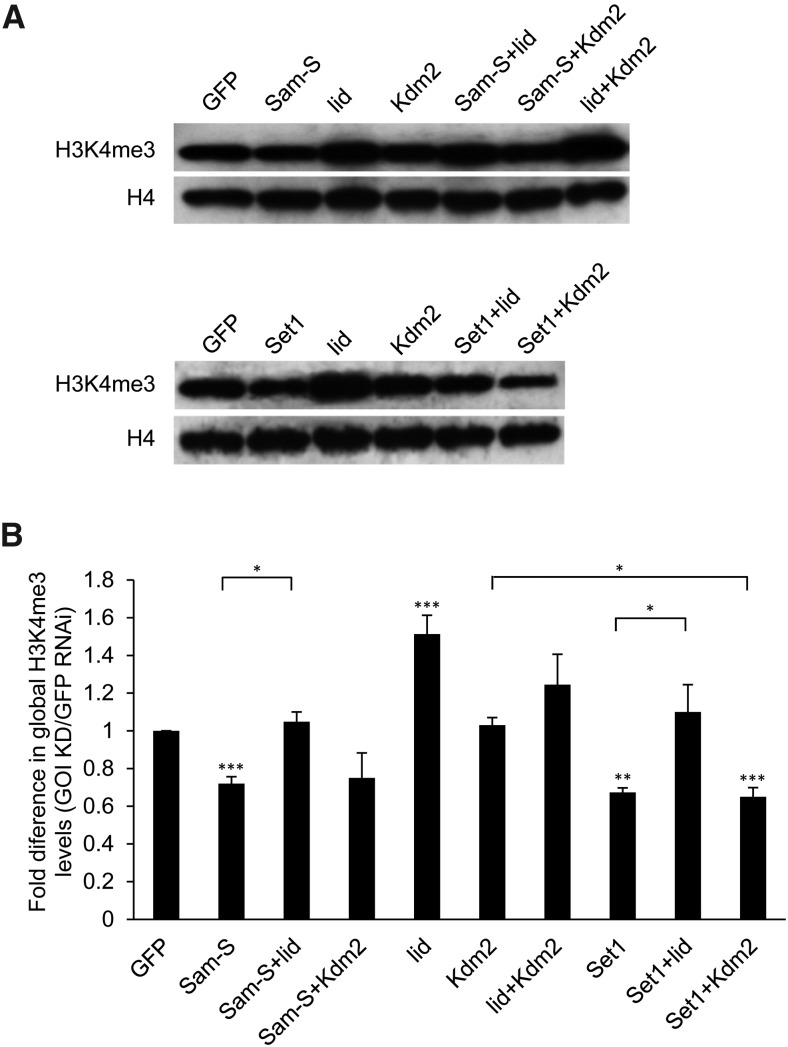
Global H3K4me3 levels in S2 cells are regulated by the methionine metabolic enzyme SAM-S, histone demethylase LID and histone methyltransferase SET1. (A) Whole cell extracts from RNAi-treated cells were subjected to western blot analysis for H3K4me3 or H4. (B) Western blots as shown in A were repeated with protein extracts prepared from at least three independent cultures and the results were quantified after normalization to histone H4. Error bars represent standard error of the mean. Statistically significant results comparing individual knockdown samples to the control are indicated on knockdown samples. *P*-values were also calculated between the double knockdown samples and each single knockdown sample for the two tested genes. Statistically significant results are indicated with the bars. (*) *P* < 0.05, (**) *P* < 0.01, (***) *P* < 0.001. GFP: control cells treated with dsRNA against GFP. GOI KD: cells treated with dsRNA against the gene(s) of interest.

Next, we wanted to explore the possible cooperation between enzymes involved in methionine metabolism and histone demethylases in regulating histone methylation. As described above, we focused on H3K4me3 mark. Consistent with published data (Ardehali *et al.* 2011; Mohan *et al.* 2011; Hallson *et al.* 2012), *Set1* influenced global H3K4me3 levels (Figure 7). *Sam-S* was the only tested methionine metabolic gene affecting global H3K4me3 levels (Figure 5), so we selected *Sam-S*, *Set1* as well as *lid* and *Kdm2* to investigate possible interactions in their contribution to H3K4me3 levels. We measured H3K4me3 levels by western blot analysis in S2 cells with different combinations of the knockdown factors. Consistent with published data (Eissenberg *et al.* 2007; Lee *et al.* 2007; Secombe *et al.* 2007; Lloret-Llinares *et al.* 2008), reduced LID led to increased global H3K4me3 levels (Figure 7). The role of KDM2 in histone methylation is still controversial (Lagarou *et al.* 2008; Kavi and Birchler 2009; Li *et al.* 2010; Zheng *et al.* 2014). Our results showed that decreased KDM2 did not affect global H3K4me3 levels (Figure 7), which suggests that KDM2 is not a major H3K4 demethylase. Additionally, although the data were not statistically significant, the H3K4me3 levels in double knockdown *lid* and *Kdm2* cells were intermediate between those of *lid* single knockdown and *Kdm2* single knockdown cells (Figure 7). These results indicate that KDM2 may counteract the demethylase function of LID in S2 cells. Compared with lowered global H3K4me3 levels in *Sam-S* or *Set1* knockdown cells, reduction of LID, but not KDM2, in the context of reduced SAM-S or SET1, restored global H3K4me3 levels to near control levels (Figure 7). These data further support our conclusion that KDM2 is not a major H3K4 demethylase. Moreover, these results suggest that LID controls H3K4me3 levels in opposition to SAM-S or SET1. SET1 is a histone methyltransferase, thus it is not surprising that LID, as a histone demethylase, acts in opposition. SAM-S likely affects H3K4me3 levels by influencing the amount of the methyl donor SAM. Reduction of LID may allow more H3K4 to remain methylated. These data demonstrate the effect on histone methylation by a metabolic enzyme can be countered by the action of a chromatin modifier.

## Discussion

In this study, we systematically analyzed two histone demethylases and the components of the methionine pathway including a single known histone methyltransferase in *Drosophila*. We investigated their role in the regulation of development, cell proliferation and histone methylation. We found that some enzymes involved in methionine metabolism and a demethylase affect viability and wing development in *Drosophila*. Further, all tested genes, except *Kdm2*, share similar roles in cell proliferation. Additionally, they cooperate to control histone methylation. Together, these data indicate the presence of a link between control of methionine metabolism and histone methylation to regulate multiple biological processes.

Reduction of SAM-S led to decreased global H3K4me3 and H3K9me2 in *Drosophila* S2 cells. H3K4me3 enrichment at a few tested genes was also affected by reduction of SAM-S, indicating the importance of SAM-S in regulation of gene specific histone methylation. Given that the level of methyl donor SAM is important for histone methylation (Brosnan and Brosnan 2006), it is likely that SAM-S regulates histone methylation by controlling SAM levels. Additionally, SAM-S affects the amounts of metabolites in polyamine pathway (Larsson *et al.* 1996). Given that polyamine can bind to DNA and affect chromatin conformational status (Matthews 1993), it is also possible that SAM-S affects histone methylation through general loss of polyamines. Interestingly, comparing our results with other published data (Li *et al.* 2011; Towbin *et al.* 2012), reduction of SAM-S did not affect all tested histone marks. SAM-S also does not influence the same histone mark in the same way among different species (Li *et al.* 2011; Towbin *et al.* 2012). Given the variety of the SAM binding affinity (*K*_m_) between histone methyltransferases (Xiao *et al.* 2003; Patnaik *et al.* 2004; Chin *et al.* 2005; Obianyo *et al.* 2008; An *et al.* 2011; Horiuchi *et al.* 2013), these differences are possibly due to variability of methyltransferase sensitivity to SAM levels (Katada *et al.* 2012). In this case, H3K4me3 and H3K9me2 specific methyltransferases are likely more sensitive to SAM levels compared to H3K4me2 specific methyltransferases. Alternatively, the levels of H3K4me2 methyltransferases, localized on chromatin at specific subdomains, may be different from the levels of H3K4me3 and H3K9me2 methyltransferases at those chromatin regions (Katada *et al.* 2012; Sassone-Corsi 2013). We note that SAM-S showed the most significant role in histone methylation among all tested methionine metabolic genes. One possible explanation is that SAM-S directly controls SAM levels, while other key methionine metabolic genes likely indirectly influence SAM levels through affecting the concentration of intermediates in the pathway.

Of the three tested enzymes annotated to have methyltransferase activity, CG10903, CG9666 and MT2, whose histone methyltransferase activities are unknown, only CG9666 and MT2 affected histone methylation in S2 cells. This result suggests that these two enzymes may directly methylate histones. There is, however, another possibility. CG9666 has been predicted to be an N6-adenine DNA methyltransferase based on sequence and structural information on Flybase (http://flybase.org) (St Pierre *et al.* 2014). MT2 has possible CpG DNA methyltransferase activity (Tang *et al.* 2003). Links between CpG DNA methylation and histone methylation have been established in other organisms, including plants (Tariq and Paszkowski 2004) and mammals (Rose and Klose 2014). Histone methyltransferase enzymes may be targeted to particular genes through recognition of methylated DNA. Thus, it is possible that CG9666 and MT2 regulate histone methylation via DNA methylation. The presence of CpG methylation in *Drosophila*, however, is the subject of current debate (Dunwell and Pfeifer 2014). Low levels of this DNA modification have been detected by some methodologies (Dunwell *et al.* 2013; Capuano *et al.* 2014; Takayama *et al.* 2014) but not by others (Zemach *et al.* 2010; Raddatz *et al.* 2013). In addition, N6-methyladenine was recently detected in *Drosophila* (Zhang *et al.* 2015). The role of this DNA modification as an epigenetic mark, however, needs much further study (Heyn and Esteller 2015). Determination of the substrates of these putative methyltransferases as well as the mechanisms through which they affect histone methylation will require further extensive biochemical analyses.

In our hands, LID, but not KDM2, removed H3K4me3. Interestingly, although the results were not statistically significant, the histone methylation levels in cells with double knockdown of *lid* and *Kdm2* were intermediate between the levels in *lid* single knockdown and *Kdm2* single knockdown cells. These observations suggest that decreased KDM2 may overcome the effect caused by reduction of LID. This assumption is consistent with previously published (Swaminathan *et al.* 2012) and unpublished (A. Gajan, V.L. Barnes, M. Liu, N. Saha, L.A. Pile, unpublished data) genetic studies from our laboratory, which showed that reduction of KDM2 or overexpression of LID can suppress the *Sin3A* knockdown curved wing phenotype in flies. One study, however, reported that *lid* or *Kdm2* mutants suppressed the *snr1^E1^* ectopic wing vein phenotype in *Drosophila*, although histone demethylase mutants *CG3654* and *CG8165* enhanced the *snr1^E1^* phenotype (Curtis *et al.* 2011). These data indicate that LID and KDM2 can act in opposition in some specific cases, which is an interesting area for further investigation.

Reduction of all tested enzymes, but not KDM2, led to decreased cell number in wing imaginal discs and cultured cells. Alterations in a number of different pathways could lead to this observed decrease in cell proliferation. For some genes, the changes in histone methylation levels following RNAi knockdown could directly affect expression of important cell cycle associated genes. Additionally, it is possible that disruption of the methionine metabolic pathway influences global protein synthesis, which in turn impacts cell growth rate. This is a likely possibility for those enzymes that were not linked to changes in histone methylation. The decreased cell number could also be due to apoptosis, though we did not observe substantial numbers of dead cells upon RNAi knockdown in the S2 cell growth assay. Interestingly, for all genes affecting cell proliferation, with the exception of *Set1*, we noticed that the cell growth defects in the developing wing imaginal disc cells were much more pronounced compared to defects observed in cultured cells. It is possible that there is a stronger requirement for this pathway and these histone modifiers during development compared to cells proliferating in the culture dish. Alternatively, this difference could be the result of cell competition, which is based on the comparison of relative cell fitness between neighboring cells (Levayer and Moreno 2013). The wild type cells grow faster than the RNAi knockdown cells, and thus the knockdown cells are eliminated during wing imaginal disc development.

RNAi knockdown of *Sam-S*, *Ahcy13* or *Cbs* resulted in lethality and cell proliferation defect in wing imaginal discs, but did not influence adult wing morphology. There are several possible reasons to explain this difference between the observed phenotypes comparing whole animal development to wing specific development. The difference is possibly due to different RNAi efficiencies when distinct drivers are utilized. Another possibility is that methionine may be supplied noncell autonomously to the RNAi knockdown wing disc cells, allowing development of a normal wing. Alternatively, these enzymes possibly play a more significant role in early stage embryogenesis relative to wing differentiation. Given that SAM-S regulated gene specific histone methylation and gene expression, it is possible that decreased histone methylation caused by *Sam-S* knockdown affects expression of genes which are critical for viability in embryogenesis, but not for differentiated wing development.

Reduction of CG10623 and MT2 affected cell proliferation in wing imaginal discs, but did not result in an abnormal wing. These contradictory results may be explained by cell competition, mentioned above (Levayer and Moreno 2013). Due to the slower growth rate compared to wild type cells, *Mt2* or *CG10623* deficient cells may be eliminated during development, leading to normal wing morphology.

Among the six tested known or potential methyltransferases and demethylases, only *CG10903*, *Set1* and *lid* knockdown flies showed defects in both cell proliferation and development. In *Drosophila*, SET1 is the main H3K4 di- and trimethyltransferase, while TRR and TRX are minor contributors for H3K4 methylation (Ardehali *et al.* 2011; Mohan *et al.* 2011; Hallson *et al.* 2012). Our results indicate that, during embryonic and wing development, TRR and TRX are not able to functionally substitute for reduction of SET1. Among all tested genes, knockdown of *Set1* led to the smallest significant decrease in cell proliferation in S2 cells and wing imaginal discs. This finding raises the possibility that SET1, TRR and TRX may be redundant in the regulation of cell proliferation in this specific cell type and wing developmental stage. Consistent with previous studies, we also demonstrate that LID is a major histone demethylase specific for H3K4me3 (Li *et al.* 2010). Therefore, SET1 and LID possibly influence cell proliferation and development via tight control of H3K4me3 levels, which in turn affects transcription of cell cycle associated genes and developmental genes. Whether TRR and TRX are able to counter the histone methylation effects due to reduction of LID, similar to the activity of SET1, is an area for future research. *CG10903* was found to have a significant role in cell proliferation and development. Expression of this gene, however, is quite low in S2 cells and during development (Graveley *et al.* 2011). While we do not have a definitive reason to explain how reduction of a gene with low RNA expression results in an observable phenotype, we note that other important developmental genes show the same pattern. For example, expression of *Pan* is low in S2 cells and during development (Graveley *et al.* 2011) and yet its reduction leads to heart development defects (Casad *et al.* 2012). Collectively, the results presented here indicate that methionine metabolism and histone methylation are critical for *Drosophila* development.

In conclusion, our findings demonstrate function and relationships of methionine metabolic enzymes and histone modifiers in regulating histone methylation. Our results reveal a role of these enzymes in influencing development and cell proliferation, which confirms the idea that metabolism and epigenetics can control key biological processes. Given that the changes of major metabolites and histone modifications are frequently observed in cancers (Katada *et al.* 2012), it is very important to understand the interaction between nutrient pathways and epigenetics in regulation of biological processes. Because the metabolic pathways and histone modifying enzymes are conserved between flies and higher eukaryotes, *Drosophila* is a good model system to use to address these questions.

## 
